# Knee Viscosupplementation: Cost-Effectiveness Analysis between Stabilized Hyaluronic Acid in a Single Injection versus Five Injections of Standard Hyaluronic Acid

**DOI:** 10.3390/ijms18030658

**Published:** 2017-03-17

**Authors:** Francisco J. Estades-Rubio, Alvaro Reyes-Martín, Victor Morales-Marcos, Mercedes García-Piriz, Juan J. García-Vera, Macarena Perán, Juan A. Marchal, Elvira Montañez-Heredia

**Affiliations:** 1 Department of Orthopedic Surgery and Traumatology, Virgen de la Victoria University Hospital, Málaga E-29010, Spain; fjestades@hotmail.com (F.J.E.-R.); mgp_jam@hotmail.com (M.G.-P.); juanjogv965@gmail.com (J.J.G.-V.); 2 Department of Orthopedic Surgery and Traumatology, Hospital Regional Universitario Carlos Haya, Malaga, Málaga E-29010, Spain; zambua@hotmail.com; 3 Department of Orthopedic Surgery and Traumatology, Hospital Quirónsalud Málaga, Málaga E-29004, Spain; victortrauma@yahoo.es; 4 Department of Health Sciences, University of Jaén, Jaén E-23071, Spain; mperan@ujaen.es; 5 Biopathology and Regenerative Medicine Institute (IBIMER), Centre for Biomedical Research, University of Granada, Granada E-18071, Spain; jmarchal@ugr.es; 6 Department of Human Anatomy and Embryology, Faculty of Medicine, University of Granada, Granada E-18016, Spain; 7 Biosanitary Institute of Granada (ibs. GRANADA), University Hospitals of Granada-University of Granada, Granada, Granada E-18016, Spain; 8 Instituto de Investigación Biomédica de Málaga (IBIMA), Málaga E-29010, Spain

**Keywords:** knee osteoarthritis, hyaluronic acid, Stabilized hyaluronic acid (NASHA)

## Abstract

Given the wide difference in price per vial between various presentations of hyaluronic acid, this study seeks to compare the effectiveness and treatment cost of stabilized hyaluronic acid (NASHA) in a single injection with standard preparations of hyaluronic acid (HA) in five injections in osteoarthritis (OA) of the knee. Fifty-four patients with knee osteoarthritis (Kellgren–Lawrence Grade II and III) and the Western Ontario and McMaster Universities Arthritis Index (WOMAC) pain score greater than 7, with a homogeneous distribution of age, sex, BMI, and duration of disease, were included in this study. Patients were randomized into two groups: Group I was treated with NASHA (Durolane^®^) and Group II with HA (Go-ON^®^). Patient’s evolution was followed up at the 1st, 2nd, 4th, 8th, 12th, and 26th week after treatment. A statistically significant improvement in WOMAC score was observed for patients treated with NASHA versus those who received HA at Week 26. In addition, the need for analgesia was significantly reduced at Week 26 in the NASHA-treated group. Finally, the economic analysis showed an increased cost of overall treatment with HA injections. Our data support the use of the NASHA class of products in the treatment of knee OA.

## 1. Introduction

Osteoarthritis (OA) of the knee is a disabling condition with a high prevalence. The burden is not limited to pain and reduced quality of life for patients but also generates high and extra hospital costs [1,2,3,4].

OA treatment depends on the natural progression of the disease and can be divided into two main stages. The first stage is characterized by the use of different alternative treatments to control primary OA symptoms based on several criteria such as the severity of symptoms and clinical response [5,6,7]. These treatments include (i) non-steroidal anti-inflammatory agents (NSAIDs); (ii) short-term options for the management of OA pain such as slow acting drugs (SYSADOAS), paracetamol, and opioids, among others; and (iii) viscosupplementation (VS) by intra-articular injections of hyaluronic acid (HA). The use of VS implies a higher short-term cost but leads to savings in the medium and long term by delaying surgery. When the pain is so severe that it hinders the patient’s daily life, the treatment progresses to the second stage, which consists in therapeutic surgery by prosthesis implantation [7].

There are different commercial preparations for VS of the knee and other joints, many of them based in HA. Its mechanism of action is similar in all cases, performing a function of “lubrication” by reducing the friction of the joint. It is still a matter of discussion as to whether the injection also improves the biological activity and vitality of the articular cartilage [3].

The degradation pathway of these preparations is orchestrated by hyaluronidases [4], enzymes secreted by the chondrocytes, and is common for all commercial hyaluronic acid products. However, there are important differences between HA compounds that depend on the synthesis process; some of these preparations are made synthetically through biotechnology, while others are derived from animals. HA products also differ in molecular weight, the life average in the joint, as well as other important aspects, such as the price, which can have a direct economic impact on the health care system.

Stabilized hyaluronic acid (NASHA) is a new generation of common HA preparations, is originated from non-animal sources, and has a long half-life and high density. The use of NASHA to treat osteoarthritis processes would reduce the number of injections from 5 to 3, or when common HA is used, to only one. At the same time, the risk associated with a greater number of punctures (septic arthritis, etc.) is also reduced. The efficacy of both HA and NASHA have been proved by numerous studies [8,9,10,11,12], but their effectiveness related to economic implications have never been compared with each other.

## 2. Results

### 2.1. Economic Study

Here, we first estimated the economic cost of both treatments on the date on which the study was conducted. Table 1 summarizes the consumable expense and cost of the health employee for each injection.

The total cost of each infiltration procedure was 11.80 euros. Treatment with HA required five injections; if each vial cost 108 euros, the final HA treatment amounted to 167 euros. On the other hand, the overall treatment with NASHA was 152 euros. Although we focus our economic analysis only in the health system cost, we cannot deny the economic impact of treatment for the patient. It is obvious that five injections imply an increment in the total expenditure of patients in terms of displacement, loss of working days, etc.

### 2.2. Baseline Characteristics of Patients

All subjects provided a detailed medical history and underwent physical examination. In the group of patients treated with NASHA (Durolane^®^), the average age was 52.9 years, and the result on the WOMAC scale was 80 points. The average body mass index was 30, and patients had suffered the disease for an average of 25.1 months. In the group of patients treated with HA (Go-ON^®^), the average age was 54.6 years, the BMI was 32.1, and the time of disease progression was 21.9 months. The functional score for the WOMAC scale was 83.4 (Table 2).

Table 3 shows a summary of the previous medical history of the patients, it can be appreciated that both patient’s groups were homogeneous. Although there were some minor differences in the number of patients that had been previously treated with one or another treatment or had had previous surgery, it was assumed that, since it is impossible to have two patients with an identical medical history, those slight differences are implicit and acceptable in any study that implies patients. Of the patients included in the study, 79.63% had osteoarthritis degree II according to the Kelgren scale, 40.74% were treated with Durolane^®^, and 38.89% were treated with Go-ON^®^ (Table 3).

### 2.3. Patient Evolution after Treatment

Table 4 shows patient evolution over 1, 2, 4, 8, 12, and 26 weeks with respect to baseline in the overall WOMAC score, pain, stiffness, and functional limitation. None of the variables analyzed have a normal distribution. Quantitative variables are expressed as medians with Quartiles 25 and 75.

Statistically significant differences between WOMAC scores from baseline and those from Week 26 were appreciated among treatments (Table 4). Patients injected with NASHA (Durolane^®^) showed a decrease in WOMAC score compared to those treated with HA (Go-ON^®^).

No statistically significant differences in the variations of mobility in the groups receiving NASHA compared to those receiving HA were found (Table 5).

Finally, patients treated with NASHA showed a significant reduction in medication (paracetamol, at a maximum dose of 1000 mg/day together with ibuprofen 600 mg if needed) after infiltration when compared with HA-treated patients (*p* = 0.006).

## 3. Discussion

The efficacy of treatment of gonarthrosis by HA viscosupplementation has been studied before. Different approaches such as radiological assessment and WOMAC function scale evaluation have been employed to measure knee osteoarthritis progression [13,14,15]. So far, seven meta-analyses have been conducted to analyze the effectiveness of this treatment; five of them concluded that the use of this therapy was beneficial for the patients [16,17,18,19,20,21], while two studies did not show any significant effect after intra-articular therapy with HA [22,23]. In this respect, it is worth pointing out that nine out of ten therapeutic guidelines for the treatment of knee OA contains recommendations for HA viscosupplementation [24].

Similar beneficial effects were reported after comparison between HA versus glucocorticoids injection; nevertheless, HA influence, although starting later, showed a more lasting effect [25]. A trial conducted in 2004 [12] showed no statistically significant differences between NASHA regarding placebo administration, although authors claimed that a more extended study was needed.

A recent systematic review of 74 randomized controlled studies on the use of intra-articular HA therapy knee OA [26] has recommended the realization of multicenter clinical trials in order to obtain further evidence of the effectiveness of various HA preparations and to determine the most suitable products and parameters such as range of molecular weight, concentration, and volume.

The aim of the present study was to evaluate the cost efficiency of two intra-articular HA preparations. Following patients’ evaluations via the WOMAC functional scale, we found statistically significant differences at Week 26 in favor of NASHA. Patients treated with NASHA showed pain improvement, resulting in a decreased need for extra medication, compared to those treated with HA. Although significant, this evident superiority in the efficacy of NASHA versus HA should be confirmed by other clinical studies.

Available pharmaco-economic studies demonstrate that HA is a cost-effective alternative to conventional treatments and has a significant impact on improving the quality of life of patients with OA [27]. We highlight the fact that HA viscosupplementation reduce health system economic burden by delaying the implantation of a prosthetic knee.

We have compared the effectiveness of HA versus NASHA and concluded that, although both preparations (Go-ON^®^ and Durolane^®^) improve joint range of motion with no statistically significant difference between then, NASHA administration enhanced WOMAC parameters and reduced the need for analgesia when compared with HA administration.

## 4. Materials and Methods

### 4.1. Patients Recruitment

Patients with symptomatic knee OA (Kellgren–Lawrence Grades II and III) were selected based on the inclusion/exclusion criteria shown in Table 6.

In the event that a patient had bilateral knee OA and both knees met the inclusion criteria, only the more symptomatic knee, with the highest score on the WOMAC pain subscale, was treated.

The protocol was planned and carried out in agreement with the Declaration of Helsinki. Patients were recruited and the study was conducted at University Hospital “Virgen de la Victoria” (Malaga, Spain) after approval by the ethics committee of the Virgen de la Victoria Hospital and AEMPS (25/11/2011_trauma). Patients signed the informed consent and were always evaluated by the same physician, who assessed the inclusion criteria. Radiographs were examined and classified according to the Kellgren and Lawrence scale. The flexion-extension arc of the knee was measured together with knee pain evaluated by the WOMAC scale value, reflecting the subject’s baseline.

### 4.2. Randomized Study

Patients were divided into two groups in a randomized fashion. Group I (*n* = 27) was treated with a single injection of NASHA (Durolane^®^ Laboratorio Zambon); Group II (*n* = 27) was treated with five injections of HA (Go-ON^®^ Laboratorio Rottapharm). Injections were performed under aseptic conditions in the outer portion of the subcuadricipital fornix, on one occasion in the group of patients treated with NASHA and five times in the HA group with a weekly interval.

Following viscosupplementation, all patients were advised to avoid NSAIDs and SYSADOA, but paracetamol, at a maximum dose of 1000 mg/day together with ibuprofen 600 mg (if needed), was allowed for pain management. Subsequent visits were performed at Weeks 1, 2, 4, 8, 12, and 26 from the first infiltration to evaluate patients’ evolution using the same parameters of joint mobility and pain. The need for analgesics throughout the period of the study was also recorded.

The primary endpoint of effectiveness was evaluated based on the WOMAC index, pain (A), stiffness (B), and fitness of the patient (C). Each patient received a WOMAC questionnaire accompanied by an instruction leaflet for self-reporting before starting the study and a new questionnaire on each successive visit for monitoring.

Other variables studied were age, sex, joint mobility, degree of OA, body mass index (BMI), previous treatments for OA, duration of disease, previous surgery in the joint, presence of joint effusion, and need for medication weekly.

Possible immediate adverse effects such as swelling, redness, or pain post-infiltration and the late, synovitis, or trophic, changes in tegument were also scored.

In addition, the costs generated by each treatment group were analyzed to find out which treatment was most advantageous from an economic point of view.

### 4.3. Statistical Analysis

To analyze differences between continuous variables in two independent groups, a Student’s *t* test was applied for two independent samples with normal distribution, tested by the Shapiro–Wilk. For non-normal distribution samples, a Mann–Whitney U test was applied. A value of *p* < 0.05 was considered significant. Data are presented as the mean ± standard error of the mean (SEM).

## 5. Conclusions

In conclusion, our study supports the use of the NASHA class of products in the treatment of knee OA and found a slight improvement in the economic impact on the healthcare system.

## Figures and Tables

**Table 1:** Total cost for each injection.

Elements Needed for the Injection	Cost (€)
Needle 40/80	0.01 €
Scandinibsa^®^ vial 2% 10 mL (eventually)	0.52 €
Gauze swab with Betadine^®^	0.26 €
Sterile surgical dressing	0.30 €
Sterile cloth eyes	0.10 €
Pair of sterile latex Hartmann	0.60 €
NASHA, Durolane^®^	140 €
HA, Go-On^®^	108 €
Nurse (estimation 10 min per injection)	4.16 €
Physician (estimation 10 min per injection)	5.86 €

**Table 2:** Patient data record on the first visit. Different letter within the same row stands for significant differences (*p* < 0.05, One way ANOVA).

Baseline Characteristics of the Patient	NASHA	HA
Age	52.9 ^a^ ± 13.9	54.6 ^a^ ± 10.5
BMI	30.0 ^b^ ± 4.5	32.1 ^a^ ± 2.4
Duration of disease	25.1 ^a^ ± 14.2	21.9 ^a^ ± 7.6
Weekly medication	4.0 ^a^ ± 1.1	4.4 ^a^ ± 1.6
Mobility	102.0 ^a^ ± 10.8	98.3 ^a^ ± 11.2
WOMAC pain score	16.2 ^a^ ± 4.4	18.2 ^a^ ± 4.4
WOMAC stiffness score	7.1 ^a^ ± 2.2	7.0 ^a^ ± 2.0
WOMAC functional limitation score	56.7 ^a^ ± 16.7	57.6 ^a^ ± 15.5
WOMAC average physical functioning	80.0 ^a^ ± 22	83.4 ^a^ ± 20.8

**Table 3:** Patient distribution according to sex, previous treatment, degree of osteoarthritis, hydrarthrosis, and previous surgery.

Patient Information		NASHA		HA	
		*n*	%	*n*	%
Sex	Male	13	24.07	16	29.63
	Female	14	25.93	11	20.37
Previous treatments	AINE^1^	17	31.48	21	38.89
	AINE, CP^2^	8	14.81	2	3.70
	AINE, CTC^3^	2	3.70	0	0.00
	CTC, AINE	0	0.00	4	7.41
Osteoarthritis degree	Degree I	0	0.00	0	0.00
	Degree II	22	40.74	21	38.89
	Degree III	5	9.26	6	11.11
Hydrarthrosis	No	22	40.74	22	40.74
	Yes	5	9.26	5	9.26
Previous surgery	Arthroscopy	3	5.56	0	0.00
	Fracture	1	1.85	0	0.00
	Meniscectomy	5	9.26	10	18.52
	No	17	31.48	17	31.48
	Yes	1	1.85	0	0.00

AINE^1^: nonsteroidal anti-inflammatory drugs; CP^2^: Chondroprotective, oral treatment with preparations containing glucosamine or chondroitin sulfate; CTC^3^: Corticoesteroids, intra-articular injection of corticosteroids such as Triamcinolone Acetonide (Trigon^®^).

**Table 4:** Patients’ parameter conditions evaluation after 26 weeks of treatment with NASHA or HA.

Patients’ Parameter Conditions	1st Week			2nd Week			4th Week			8th Week			12th Week			26th Week		
	NASHA	HA	*p*	NASHA	HA	*p*	NASHA	HA	*p*	NASHA	HA	*p*	NASHA	HA	*p*	NASHA	HA	*p*
WOMAC	1 (−3.10)	1 (−3.2)	0.5	6 (−1.12)	2 (0.17)	0.81	14 (3.18)	2 (1.15)	0.03	21 (7.38)	6 (4.16)	0.01	24 (8.36)	10 (6.17)	0.0193	24 (13.34)	10 (6.13)	0.01
Pain	0 (−1.2)	1 (0.3)	0.5	2 (0.2)	2 (0.5)	0.28	2 (0.4)	2 (0.4)	0.96	5 (3.7)	3 (1.8)	0.42	6 (3.9)	4 (3.9)	0.18	6 (5.9)	3 (2.7)	0.01
Stiffness	0 (0.1)	0 (−1.1)	0.4	1 (1.2)	0 (0.1)	0.03	1 (1.3)	1 (0.1)	0.01	3 (2.4)	1 (1.2)	0.01	3 (2.4)	2 (1.3)	0.01	4 (3.5)	2 (1.3)	0.01
Functional limitation	1 (−1.8)	0 (−2.1)	0.09	5 (−2.10)	0 (−2.9)	0.40	9 (2.17)	2 (−2.8)	0.09	16 (2.27)	2 (0.11)	0.01	18 (5.23)	3 (−1.11)	0.01	15 (5.21)	3 (0.10)	0.01

**Table 5:** Mobility assessment in patients treated with NASHA versus HA treatment.

Patient Identification Code	NASHA Median (p25, p75)	HA Median (p25, p75)	*p*
difmovs1	0 (0,0)	0 (0,0)	0.1473
difmovs2	0 (0,0)	0 (−5,0)	0.3548
difmovs4	−5 (−10,0)	−5 (−5,0)	0.4883
difmovs8	−10 (−10,−5)	−5 (−10,−5)	0.4030
difmovs12	−10 (−20,−5)	−10 (−10,0)	0.7423
difmovs26	−10 (−20,−5)	−5 (−15,0)	0.2426

**Table 6:** Inclusion and exclusion criteria.

Inclusion Criteria	Exclusion Criteria
Kellgren–Lawrence Grade II and III	Known hypersensitivity to HA
WOMAC pain score greater than 7 Pain in the knee for more than 5 days a week in the previous 3-month	Known hypersensitivity or contraindication to paracetamol or ibuprofen
Pain in the knee for more than 5 days a week in the previous 3-month	Intra-articular injection of corticosteroids in the previous 3-months
Patients able to walk at least 50 meters unassisted Patients with an active lifestyle	Arthroscopy or local surgical procedure in the previous 3 months Systemic corticosteroids in the previous 3 months Rheumatoid arthritis or other systemic inflammatory process Pathological skin on the puncture site or close to it Pregnant or breastfeeding women or in childbearing age not using contraception

## Abbreviations

NASHA	Stabilized hyaluronic acid
HA	Hyaluronic acid
OA	Osteoarthritis
WOMAC	Western Ontario and McMaster Universities Arthritis Index
NSAIDs	Non-steroidal anti-inflammatory agents
SYSADOAS	Slow acting drugs
VS	Viscosupplementation
AINE	Nonsteroidal anti-inflammatory drugs
CTC	Corticoesteroids
CP	Chondroprotective
SEM	Standard error of the mean
